# In Vitro Evaluation of Antidiabetic Potential of *Cleistocalyx nervosum* var. *paniala* Fruit Extract

**DOI:** 10.3390/plants12010112

**Published:** 2022-12-26

**Authors:** Suttida Chukiatsiri, Nattakarn Wongsrangsap, Siriluk Ratanabunyong, Kiattawee Choowongkomon

**Affiliations:** Department of Biochemistry, Faculty of Science, Kasetsart University, Bangkok 10900, Thailand

**Keywords:** *Cleistocalyx nervosum* var. *paniala*, type 2 diabetes mellitus, α-amylase and α-glucosidase inhibitors, glucose uptake, lipid accumulation

## Abstract

Diabetes mellitus is a complex global public health condition. Medicinal plants are significant resources in the research of alternative new drug active compounds. *Cleistocalyx nervosum* var. *paniala* (*C. nervosum*) is an indigenous berry fruit widely grown in Southeast Asia. The fruit of *C. nervosum* exhibit various medicinal properties and health benefits. This study aimed to investigate antidiabetic properties of *C. nervosum* fruit extract by in vitro assays and in vitro models. *C. nervosum* fruit extracted using three different solvents (hexane, ethanol, and distilled water) were tested for α-amylase and α-glucosidase inhibitory activities, followed by glucose uptake in HepG2 and L6 myoblasts. Lipid accumulation in 3T3-L1 cells treated with *C. nervosum* fruit extracts was then examined. The results revealed that ethanolic extract of *C. nervosum* fruit showed better inhibition against α-amylase (IC_50_ of 0.42 μg/mL) and α-glucosidase (IC_50_ of 0.23 μg/mL) compared with other extracts. Furthermore, ethanolic extract showed higher glucose uptake potential than the standard antidiabetic drug, metformin, in HepG2 cells. The ethanolic extracts resulted in enhanced glucose utilization in L6 myoblasts compared to untreated control. All extractions showed no significantly increased lipid accumulation in 3T3-L1 cells compared to the untreated control cells. The investigation confirmed that the ethanolic extract exhibited the highest antidiabetic activity among all extracts. These results imply that *C. nervosum* fruit extract has antidiabetic properties and therefore they may be used as useful therapeutic agents for treating diabetes.

## 1. Introduction

Diabetes mellitus is one of the major causes of morbidity and mortality worldwide [1]. Diabetes mellitus is a non-communicable disease which is a serious complex multifactorial disorder due to deficiency in insulin secretion or defect in insulin action [2]. Type 2 diabetes, characterized by hyperglycemia, is a public health problem acknowledged as one of the major economic burdens. If left untreated, it can lead to severe complications, including hyperlipidemia and oxidative stress [3]. Even though numerous oral hypoglycemic agents that are currently used for the treatment of type 2 diabetes are facing limited efficacy and tolerability, the use of natural products with antidiabetic activity has significantly increased. Herbal drugs are prescribed widely because of their effectiveness, less side effects, and relatively low cost [4,5,6].

*Cleistocalyx nervosum* var. *paniala* (*C. nervosum*), commonly known as Ma kiang, belongs to the Myrtaceae plant family. This plant with indigenous berry fruit is widely distributed and cultivated in Southeast Asian countries, especially in the northern part of Thailand [7]. *C. nervosum* is used in traditional medicine [8]. The fruits, seeds, flower buds, and leaves of *C. nervosum* exhibit various medicinal properties and health benefits such as antioxidant and antiaging properties, antimutagenicity and anticarcinogenic activities, antiheavy metal toxicity, antimicrobial activities, immune enhancement, and neuroprotective property [9,10,11,12,13,14]. However, the antidiabetic activity of *C. nervosum* fruit extracts has rarely been reported.

*C. nervosum*, an edible fruit, contains a high amount of anthocyanins [15]. Recent studies have suggested that the consumption of anthocyanins lowers the risk of cancer, cardiovascular disease, and diabetes, due to their properties of antioxidant and anti-inflammatory activities [16]. Moreover, the polyphenols in plants have been reported to play an important role in the mechanism for regulating the activities of carbohydrate-hydrolyzing enzymes [17]. We therefore hypothesized that *C. nervosum* fruit might provide antidiabetic potential. However, there is not much research on antidiabetic effects of *C. nervosum* fruit and most of this fruit has not been scientifically validated for its efficacy in the treatment. Therefore, this study aimed to evaluate antidiabetic activities of *C. nervosum* fruit extract using various in vitro assays and in vitro models.

## 2. Materials and Methods

### 2.1. Extraction of Cleistocalyx nervosum var. paniala Fruits

Dried fruits of *Cleistocalyx nervosum* var. *paniala* were weighted and soaked in various solvents, e.g., distilled water, ethanol, and hexane (Sigma-Aldrich, Inc., Darmstadt, Germany), at 37 °C for 24 h. At the end of incubation time, all samples were blended and centrifuged for collecting supernatants. All clear supernatants were dehydrated at 80 °C until solvent residues were eliminated. Extraction was then weighted and stored at 4 °C until analysis. The yield of extracts can be determined in percentage using the formula:

% Yield = (Dry weight of extract/Dry weight of plant material) × 100

### 2.2. Assay of α-Amylase Inhibition In Vitro

α-Amylase inhibitory activity was assayed according to the procedure described by Odeyemi et al. [18] with a slight modification. α-Amylase activity was determined using soluble starch (1%) as a substrate in 0.02 mol/l sodium phosphate buffer (pH 6.9) (Sigma-Aldrich, Inc., Darmstadt, Germany). *Cleistocalyx nervosum* var. *paniala* extracts with concentration 0.1 mg/mL, 0.25 mg/mL, 0.5 mg/mL, and 1 mg/mL were mixed with substrate solution made up to a total volume of 150 µL; 10 μL of α-amylase solution (1 unit/mL/min) (Sigma-Aldrich, Inc., Darmstadt, Germany) was added. After incubation at 25 °C for 30 min, 30 μL of dinitrosalicylic acid reagent (Sigma-Aldrich, Inc., Germany) was added and incubated at 90 °C for 5 min. The absorbance was measured at 540 nm. Acarbose (α-amylase inhibitor) (Bio Basic Inc., Markham, ON, Canada) was used as a positive control. The assay was performed in triplicate. The percentage of inhibition was calculated using the following formula:

% Inhibition activity = [(A_control (without extract)_ − A_sample_)/A_control (without extract)_] × 100

where A is the absorbance reading measured at 540 nm.

The IC_50_ value was defined as the concentration of the compound required to inhibit 50% of the α-Amylase activity under the assay conditions.

### 2.3. Assay of α-Glucosidase Inhibition In Vitro

The inhibition of α-glucosidase assay is a modification of the method previously described by Sun et al. [19]. *Cleistocalyx nervosum* var. *paniala* extracts with concentration 0.1 mg/mL, 0.25 mg/mL, 0.5 mg/mL, and 1 mg/mL were mixed with 0.1 mol/l potassium phosphate buffer (pH 6.9) (Sigma-Aldrich, Inc., Darmstadt, Germany) made up to total volume of 150 µL; 10 µL of α-glucosidase solution (1 unit/mL/min) (Sigma-Aldrich, Inc., Darmstadt, Germany) was added. The mixer was incubated at 37 °C for 15 min. Then, 10 mL of 3 mM p-nitrophenyl-α-Dglucopyranoside (PNP-G) (Sigma-Aldrich, Inc., Darmstadt, Germany) was added, and the mixture was re-incubated at 37 °C for 10 min. The reaction was terminated by the addition of 30 mL of 0.1M sodium carbonate (Sigma-Aldrich, Inc., Darmstadt, Germany). The amount of released product (p-nitrophenol) was measured at 405 nm using a UV spectrophotometer to estimate the enzymatic activity. Acarbose (α-glucosidase inhibitor) (Bio Basic Inc., Markham, ON, Canada) was used as a positive control. The assay was performed in triplicate. The percentage of inhibition was calculated using the following formula:

% Inhibition activity = [(A_control (without extract)_ − A_sample_)/A_control (without extract)_] × 100

where A is the absorbance reading measured at 405 nm.

The IC_50_ value was defined as the concentration of the compound required to inhibit 50% of the α-Glucosidase activity under the assay conditions.

### 2.4. Cell Culture

HepG2 (ATCC HB-8065), L6 myoblasts (ATCC CRL-1458), and 3T3-L1 preadipocytes (ATCC CL-173) were cultured with Dulbecco’s Modified Eagle’s Medium (DMEM) (Biochrom GmbH, Berlin, Germany) supplemented with 10% fetal bovine serum (Biochrom GmbH, Germany), and 100 U/mL penicillin and streptomycin (Biochrom GmbH, Berlin, Germany). All cells were routinely cultured in a humidified atmosphere with 5% CO_2_ at 37 °C.

### 2.5. Cytotoxicity Assay

HepG2 cells or L6 myoblasts were seeded at appropriate number of cells in a 96-well plate before experiment starting. All *Cleistocalyx nervosum* var. *paniala* fruit extracts should be determined working concentration (10 mg/mL) by diluting with PBS (Biochrom GmbH, Berlin, Germany). After cell attachment, the cells were then replenished with complete media containing individual *Cleistocalyx nervosum* var. *paniala* extractions continually incubated for 72 h. To assess cytotoxicity, the cells were replenished with complete media containing MTT solution (Sigma-Aldrich, Inc., Darmstadt, Germany) (0.5 mg/mL final concentration) and continually incubated for 3 h; 0.5% DMSO (Sigma-Aldrich, Inc., Darmstadt, Germany) was added to all samples to solubilize the formazan crystals, and then measured by using microplate reader at 570 nm measurement wavelength and 630 nm reference wavelength. This experiment was performed in triplicate and repeated 3 times. Mean values ± SD for each concentration was determined. Calculation of cell viability (in percentages, %) was shown as ratio of absorbance (A_570 nm_) in treated cells relative to absorbance in untreated cells (A_570 nm_) [20]. The percentage of viability was calculated using the following formula:

% viability = 100 × [A_sample_/A _control_]

where A is the absorbance reading measured at 570 nm.

### 2.6. Glucose Utilization Experimental Procedure on HepG2

The glucose utilization in HepG2 cells was determined by the method described by van de Venter et al. [21] with a slight modification. The HepG2 cells were seeded into 96-well culture plates at a density of 6000 cells/well and cultured with DMEM containing 10% FBS in a humidified incubator with 5% CO_2_ at 37 °C for 3 days. Two cell-free rows were also included to serve as blanks. On day three after seeding, without changing the medium, 10 µL of the 1 mg/mL *Cleistocalyx nervosum* var. *paniala* fruit extracts was added to each well. After 48 h incubation, the spent culture medium was removed and replaced with a 25 µl incubation buffer (RPMI medium diluted with PBS,0.1%BSA and 8 mm glucose) and further incubated for an additional 3 h at 37 °C. Metformin were used as the positive controls while the negative control (untreated) contained only the incubation buffer without extract. After incubation, 10 µL of the incubation medium was removed from each well and transferred into a new 96-well plate into which 200 µL of glucose oxidase reagent (Sigma-Aldrich, Inc., Germany) was added to determine the concentration of glucose in the medium. After 15 min of incubation at 37 °C, the absorbance was measured at 492 nm. Three independent experiments were conducted. The amount of glucose utilized was calculated as the difference between the cell-free and cell-containing wells. The percentage of glucose utilization was calculated in relation to the untreated controls.

### 2.7. Glucose Utilization Experimental Procedure on L6 Myoblasts

The glucose utilization in L6 myoblast cells was determined according to the methods described by van de Venter et al. [21]; L6 cells were seeded into 96-well culture plates at a density of 5000 cells/well and cultured with DMEM containing 10% FBS until 90% confluence. Culture medium was removed and replaced with DMEM containing 2% FBS and cultured for 3 days. Then, culture medium was removed, and the cells were then replenished with complete medium containing individual *Cleistocalyx nervosum* var. *paniala* extractions (1 mg/mL final concentration) continually incubated for 48 h. Additional wells were treated with insulin (4 µg/mL) (Sigma-Aldrich, Inc., Darmstadt, Germany) instead of the *Cleistocalyx nervosum* var. *paniala* extract to serve as a positive control. To assess glucose utilization, the spent medium was removed and replaced with a 25 µL incubation buffer containing RPMI medium (Biochrom GmbH, Berlin, Germany) diluted with PBS, 0.1% BSA, and 8 mM glucose, and incubated for a further 3 h at 37 °C. After the incubation period, 5 µL of the incubation medium was removed from each well and placed into a new 96-well plate. Then, 200 µL glucose oxidase reagent was added per well and incubated for 15 min at 37 °C. To determine the concentration of glucose in the medium, the absorbance was measured at 520 nm. Three independent experiments were conducted. The amount of glucose utilized was calculated as the difference between the cell-free and cell-containing wells. The percentage of glucose uptake was calculated in relation to the untreated controls.

### 2.8. Lipid Accumulation in 3T3-L1 Preadipocytes

Lipid accumulation in 3T3-L1 preadipocytes was determined according to the method described by Odeyemi et al. [22]; 3T3-L1 cells were seeded into 96-well culture plates at a density of 5000 cells/well and cultured in DMEM containing 10% FBS and 1% penicillin−streptomycin solution at 37 °C under 5% CO_2_ atmosphere and allowed to grow until 100% confluence was reached. Two days after confluence, culture medium was removed, and cells were then replenished with complete medium containing individual *Cleistocalyx nervosum* var. *paniala* extractions (1 mg/mL final concentration) or positive control (rosiglitazone; 0.4 µg/mL) continually incubated for 48 h. Cells were then cultured for an additional eight days. Then, the spent culture medium was removed, and cells were gently washed with phosphate-buffered saline, fixed with 10% formaldehyde in PBS for 1 h and then washed with 60% isopropanol. The cells were stained with 0.6% Oil Red O solution (6 mL of stock solution (0.5 g oil red dye in 100 mL isopropanol) (Sigma-Aldrich, Inc., Darmstadt, Germany) in 4 mL of distilled water)) for 15 min at 37 °C. Stained Oil Red O was also eluted with 100% isopropanol and quantified by measuring absorbance at 500 nm. A minimum of 3 independent experiments were performed, each in triplicate.

## 3. Statistical Analysis

The data were analyzed by analysis of variance (ANOVA) and the comparison of means was performed by Tukey test using GraphPad Prism 7.02 software (GraphPad Software 7825 Fay Avenue, Suite 230, La Jolla, CA 92037 USA). Data are expressed as mean ± SD of three independent experiments. The *p* values less than 0.05, 0.01 or 0.001 were considered statistically significant.

## 4. Results

Yield of the *Cleistocalyx nervosum* var. paniala fruit extract prepared using different solvents.

Effect of different solvents on extraction yield was shown in Table 1. Results showed a significant difference in the extraction yield using different solvents. Among solvents tested, distilled water resulted in the highest extraction yield (20.27%), followed by ethanol (13.39%) and hexane (8.01%).

### 4.1. α-Amylase Inhibitory Activity

The inhibitory effects of water, ethanol, and hexane extracts of *C. nervosum* on α-amylase activity were shown as inhibition concentration at 50% inhibition (IC_50_) values in Table 2. Ethanol extract showed the highest α-amylase inhibitory activity (IC_50_ = 0.42 mg/mL) than those from other samples (IC_50_ of water extraction = 0.61 mg/mL) but less than positive control, acarbose (IC_50_ = 0.09 mg/mL). The ethanol and water extracts exhibited anti-α-amylase activity in a dose-dependent manner. However, hexane extraction showed no inhibitory activity.

### 4.2. α-Glucosidase Inhibitory Activity

Table 3 shows the inhibitory effects of water, ethanol, and hexane extracts of *C. nervosum* on α-glucosidase activity. The water and ethanol extracts of *C. nervosum* exhibited a dose-dependent inhibition of α-glucosidase. Ethanol extracts exhibited the better anti-α-glucosidase activity than water and hexane extracts. The IC_50_ values of aqueous and ethanolic extraction were 0.44 mg/mL and 0.23 mg/mL, respectively. On the other hand, hexane extract exhibited no inhibitory activity against α-glucosidase. As positive control, acarbose showed lower IC_50_ (0.12 mg/mL) than all extracts.

### 4.3. Cytotoxicity

**Figure 1:**
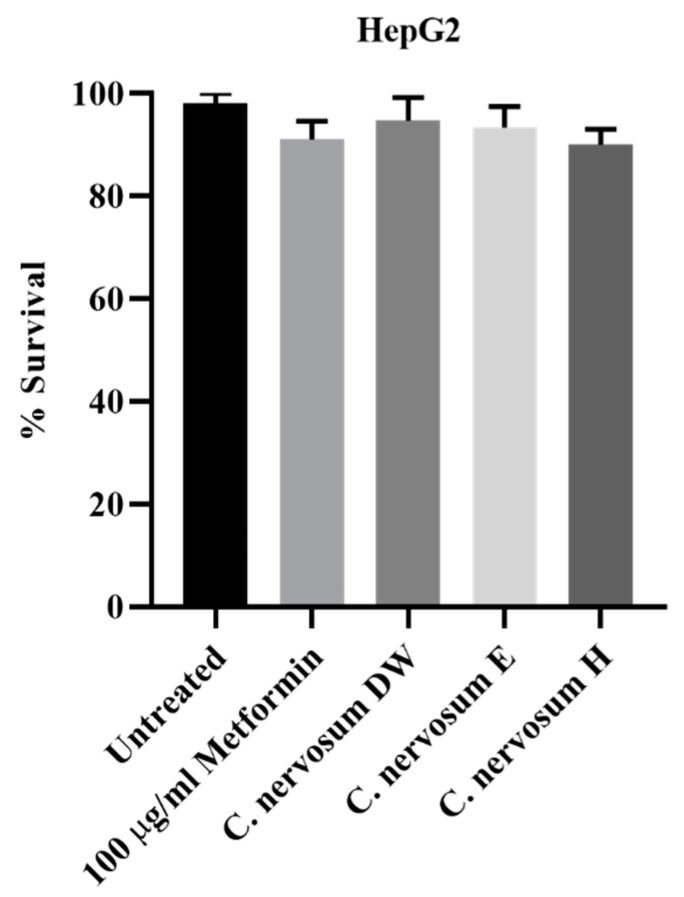
The cytotoxicity effect of water, ethanol, and hexane extracts of *C. nervosum* at concentration of 1 mg/mL on HepG2 liver cells after 72 h exposure assessed by MTT assay. Data are mean ± SD of three independent experiments. The control was cells not treated with extracts. No significant difference relative to the untreated control was noted.

The in vitro cytotoxicity of water, ethanol, and hexane extracts of *C. nervosum* at concentration of 1 mg/mL was analyzed through MTT assay against the HepG2 liver cells. The effects of all tested extracts on the viability of the HepG2 are presented in Figure S1 (Supplementary Materials). The commonly used antidiabetic drug metformin was used as reference agent. The cytotoxicity results revealed that all extracts showed no significant difference of cell death when compared to 100 µg/mL metformin (positive control) (Figure 1). Additionally, water, ethanol, and hexane extracts of *C. nervosum* exhibited without significant cytotoxic effect against HepG2 compared to untreated control.

**Figure 2:**
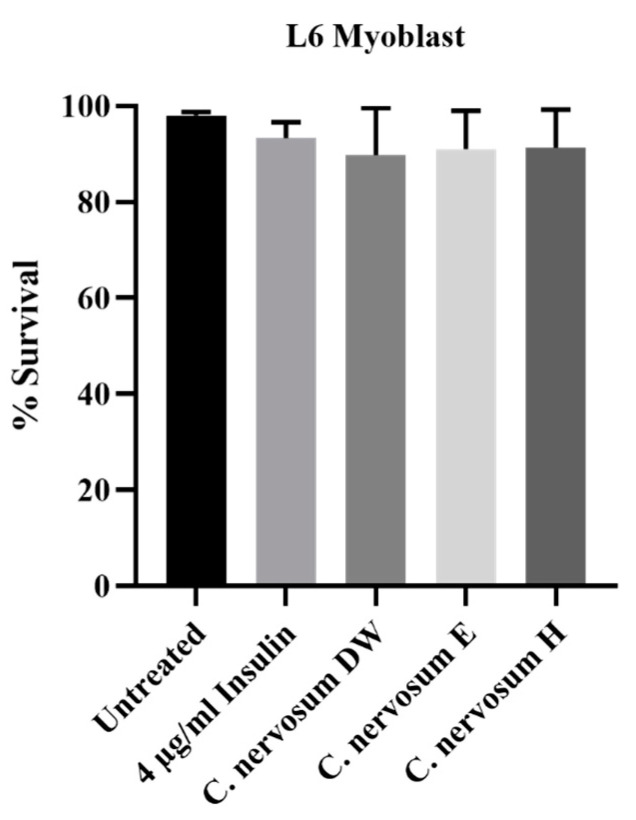
The cytotoxicity effect of water, ethanol, and hexane extracts of *C. nervosum* at concentration of 1 mg/mL on L6 myoblasts after 72 h exposure assessed by MTT assay. Data are mean ± SD of three independent experiments. The control was cells not treated with extracts. No significant difference relative to the untreated control was noted.

In addition, the cytotoxicity of *C. nervosum* extracts were determined using MTT assay in L6 myoblasts. The effects of all tested extracts on the viability of the L6 myoblasts are presented in Figure S2 (Supplementary Materials). Cell survival analyses indicated that water, ethanol, and hexane extracts of *C. nervosum* at concentration of 1 mg/mL exhibited no significant cytotoxic activity compared with 4 µg/mL insulin (positive control) and untreated control (Figure 2).

### 4.4. Glucose Utilization in HepG2

**Figure 3:**
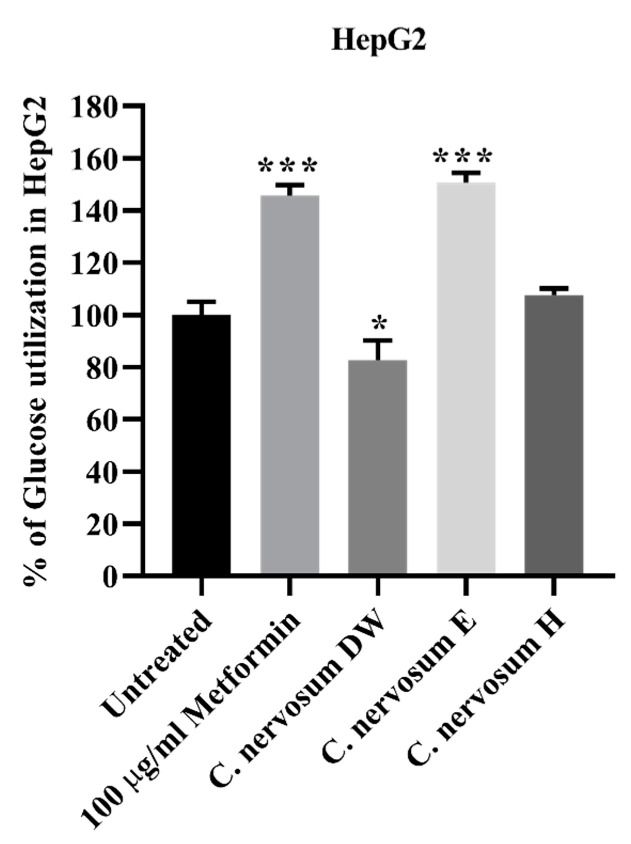
Effect of *C. nervosum* extracts on glucose utilization in HepG2 hepatocytes. Cells were treated for 48 h. Data are mean ± SD of four independent experiments. *** *p* < 0.001, * *p* < 0.05 versus the untreated control.

The results obtained for glucose uptake in HepG2 cells in the presence of *C. nervosum* extracts at 1 mg/mL are presented in Figure 3. The ethanol extracts of *C. nervosum* caused a significant higher increase in glucose uptake in HepG2 cells when compared to the untreated control. This increased glucose uptake was comparable to the presence of metformin. The hexane extracts exhibited no significant difference relative to the untreated control. On the other hand, the water extracts of *C. nervosum* showed a significant decrease in glucose uptake compared to the untreated control.

### 4.5. Glucose Utilization in L6 Myoblasts

**Figure 4:**
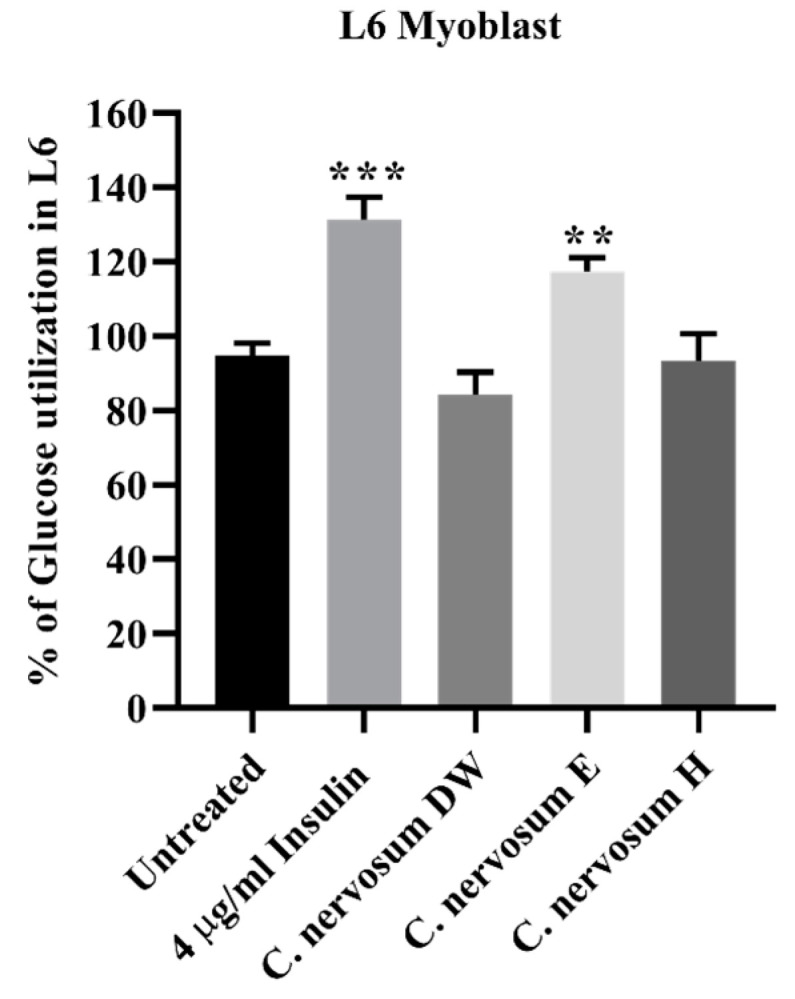
Effect of *C. nervosum* extracts on glucose utilization in L6 myoblasts. Cells were treated for 48 h. Data are mean ± SD of four independent experiments. *** *p* < 0.001, ** *p* < 0.01 versus the untreated control.

The effect of the extract of *C. nervosum* at a concentration of 1 mg/mL on L6 myoblast glucose utilization is shown in Figure 4. The results revealed that the ethanol extract of *C. nervosum* caused a significant higher increase in glucose uptake in L6 myoblasts when compared to the untreated control. However, this ethanol extraction showed lower stimulation of glucose uptake compared with insulin (4 µg/mL) used as a positive control. On the other hand, the water and hexane extracts of *C. nervosum* exhibited no significant difference relative to the untreated control.

### 4.6. Lipid Accumulation in 3T3-L1 Preadipocytes

**Figure 5:**
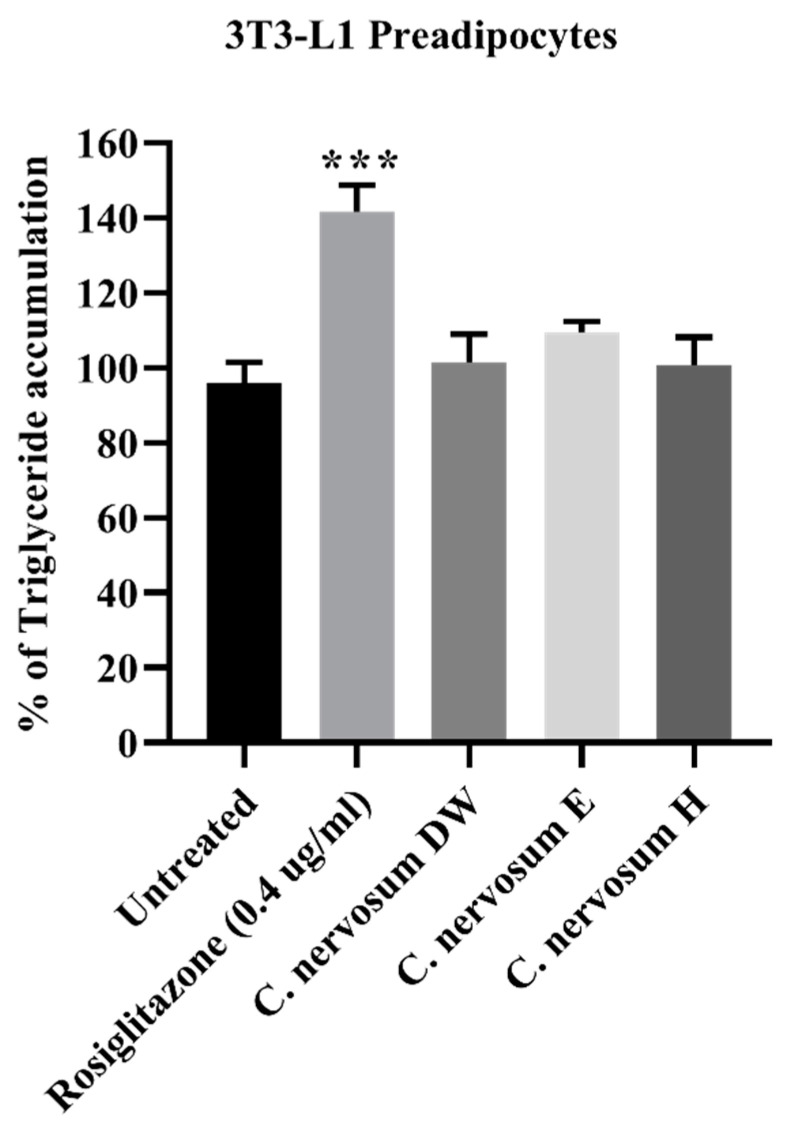
Effect of *C. nervosum* extracts on lipid accumulation in 3T3-L1 preadipocytes. Data are mean ± SD of four independent experiments. *** *p* < 0.001 versus the untreated control.

One of the severe complications caused by diabetes mellitus is hyperlipidemia. Lipid accumulation in 3T3-L1 preadipocytes was determined. Figure 5 displayed the effect of *C. nervosum* extracts at a concentration of 1 mg/mL on lipid accumulation. All three different extracts of *C. nervosum* showed no significantly increased lipid accumulation in 3T3-L1 cells compared to the untreated control cells. On the other hand, the positive control, 0.4 µg/mL rosiglitazone, exhibited significant increase in lipid accumulation in 3T3-L1 cells compared with the untreated cells.

## 5. Discussion

Diabetes mellitus is one of the largest global health emergencies of the century that affects patients’ daily life and elevates patients’ risk of developing other diseases [1]. There is a need for a safer, more effective, and less costly treatment. Plant-derived compounds provide a rich source for new drug discovery [23].

Type 2 diabetes is characterized by high concentrations of blood glucose which can cause serious complications in many organs. Therefore, the treatment of type 2 diabetes basically focuses on reducing fluctuations in blood glucose. The carbohydrate hydrolyzing enzyme inhibitors, α-amylase, and α-glucosidase inhibitors, are currently used for diabetic treatment [21]. α-Amylase is a vital enzyme of the digestive system which hydrolyzes starch into a mixture of smaller oligosaccharides which are further broken down by α-glucosidase into glucose. Thus, inhibition of α-amylase and α-glucosidase delays the breakdown of carbohydrates and decreases the postprandial blood glucose levels [24]. The inhibition of these two prominent enzymes has been found as a useful and effective strategy to lower the levels of postprandial hyperglycemia [25]. In this study, the potential antidiabetic activity of the *C. nervosum* fruit extract was investigated using various in vitro assays and in vitro models. This study demonstrated that ethanol extract of *C. nervosum* fruit had the highest inhibition of α-amylase and α-glucosidase with dose-dependent manner, compared with other extractions. The solubility of phenolic compounds is dependent on the chemical nature of the plant, as well as the polarity of the solvents used. Therefore, the type of extraction solvent, as well as the isolation procedures, may have a significant impact on the extraction yield of phenolic compounds and flavonoids from plant material [26]. In vitro studies exhibited that aqueous extract of *Eugenia operculata Roxb*. leaves showed the highest α-amylase inhibitory activity, but ethanol extract showed the highest inhibition activity of α-glucosidase [27]. Mai et al. reported that the aqueous extract of the flower bud of *Cleistocalyx operculatus* had an inhibitory effect on α-glucosidase in vitro [17]. Previous studies indicated that *C. nervosum* fruit contains a high number of phenolic compounds and flavonoids such as anthocyanin [16]. The presence of these phenolic compounds and flavonoids have been attributed to the hypoglycemic action of various plants [28]. In addition to their antioxidant effects, phenolic compounds and flavonoids have been reported to exert anti-hyperglycemic effects by inhibition of α-amylase and α-glucosidase through non-specific binding, leading to inhibition of enzyme activity, [29] and by increasing in glucose uptake through binding to glucose transporters [30]. Our results suggested that the phenolic compounds of *C. nervosum* fruit exerted hypoglycemic activity via the inhibition of α-amylase and α-glucosidase enzyme activities. However, our findings showed that the ethanol extraction *C. nervosum* fruit had lower inhibitory activity of α-amylase and α-glucosidase than acarbose (positive control). Acarbose is a commercially available α-amylase and α-glucosidase inhibitor for type 2 diabetes. It is reported to cause various side effects such as diarrhea and other intestinal disturbances, with corresponding intestinal pain and flatulence [31].

We evaluated the cytotoxicity of *C. nervosum* fruit extract on HepG2 cells and L6 myoblasts using MTT assay. This test is widely used in in vitro toxicology studies for the detection of cytotoxic and other negative effects on cell viability following exposure to test materials [20]. It is worth noting that toxicity has been one of the major concerns of plant extracts. This study clearly shows that *C. nervosum* fruit extracts were not cytotoxic to the HepG2 cells and L6 myoblasts at the tested concentrations and that the glucose uptake observed was not due to the cytotoxic effects of the tested samples. Wang et al. reported that Cleistocalyxic acid B from leaves of *Cleistocalyx operculatus* displayed cytotoxicity against HepG2 [32]. Previous study showed that *C. nervosum* fruit extract caused no significant change in mouse hippocampal neuronal HT22 cell viability compared with the non-treated control cells [33]. Therefore, the relatively low level of toxicity exhibited by the extract of *C. nervosum* fruit raises prospects that ethanol extract of *C. nervosum* could be potentially safe for the treatment.

The results obtained in this study on glucose uptake using HepG2 cells indicated that ethanolic extract of *C. nervosum* increased glucose uptake in HepG2 cells when compared to the untreated control. It also showed no significant change in glucose uptake when compared with the positive control, metformin. This suggests that the ethanolic extraction of *C. nervosum* mimics metformin by increasing glucose uptake in the liver. Metformin is currently the drug of first choice for the treatment of type 2 diabetes. Metformin is regarded as an oral anti-hyperglycemic agent because it lowers blood glucose concentrations in type 2 diabetes without causing overt hypoglycemia [34].

L6 myoblasts derived from the skeletal muscle represent a good model for glucose utilization and a primary target tissue for insulin action. They have been used extensively to study the mechanisms of glucose uptake in muscle [35]. Skeletal muscle is a major tissue involved in insulin-induced stimulation of glucose uptake [36]. Previous studies reported that 2′,4′-Dihydroxy-6′-methoxy-3′,5′-dimethylchalcone, one of the flavonoids isolated and purified from the dried flower buds of *Cleistocalyx operculatus*, did not affect glucose uptake in L6 myoblasts and glycogen synthesis in HepG2 [37]. Our findings showed that ethanol extract of *C. nervosum* fruit demonstrated promising anti-hyperglycemic potential in L6 cell line. This suggests that *C. nervosum* extracts promoted glucose utilization in skeletal muscle, and act by mechanisms that mimic insulin-mediated signaling, possibly involving increased mobilization of GLUT 4 molecules to the plasma membrane [38]. On the other hand, a mechanism of action of *C. nervosum* might be hypothesized which could be linked to activation of the insulin-signaling cascade, resulting in stimulation of GLUT 2 that facilitates the translocation of glucose into the cell [39]. Ethanolic extract of *C. nervosum* showed no potential for toxicity to HepG2 and L6 myoblasts at the concentration tested, suggesting that the observed stimulation of glucose utilization by *C. nervosum* might be a true reflection of its hypoglycemic activity.

The excessive triglyceride accumulation by the adipocyte has been linked to an in-creased risk of a variety of diseases including diabetes [40]; 3T3-L1 cells are widely used models of adipocyte function to investigate the mechanisms of action by which plant ex-tracts exert their antidiabetic effects. Most drugs used for treating diabetes cause obesity as a side effect by reducing hyperglycemia and inducing adipogenesis. Since obesity is a side effect of some antidiabetic drugs, therefore, the effect of extracts on lipid accumulation was also evaluated [41]. This study showed that all three different extracts of *C. nervosum* showed no significantly increased lipid accumulation in 3T3-L1 cells compared to the untreated control cells. On the other hand, the positive control, rosiglitazone, exhibited significant increase in adipogenesis in 3T3-L1 cells compared with the untreated cells. Rosiglitazone, a thiazolidinedione (TZD) drug, is used in the treatment of type 2 diabetes mellitus [42]. Rosiglitazone is an antidiabetic insulin-sensitizing agent that binds to the peroxisome proliferator-activated receptor γ (PPARγ) and have potent adipogenic effects on 3T3-L1 preadipocytes [43]. Hu et al. demonstrated a mechanism whereby flower bud extract of *Cleistocalyx operculatus* had paradoxical effects on lipid accumulation in 3T3-L1 cells. High concentrations of the extract (10 and 20 μM) markedly diminished lipid accumulation; however, a low concentration (2.5 μM) enhanced lipid storage in 3T3-L1 cells [37]. Our results indicated that the extracts of *C. nervosum* displayed no significantly increased lipid accumulation in 3T3-L1 adipocytes suggesting that the *C. nervosum* extract might be a good antidiabetic drug without adipogenic effect.

This study showed that ethanolic extract of *C. nervosum* fruit exhibited stronger antidiabetic activity than other extraction solvents. Due to the variety of bioactive compounds contained in plant materials and their differing solubility properties, differences in the polarity of the extraction solvents could cause a wide variation in the level of bioactive compounds in the extract [44]. Different solvents exhibited different efficacies of extraction [45]. It has been suggested that ethanolic extracts from plants used in allopathic medicine are potential sources of biological active agents [46].

## 6. Conclusions

Though several studies have reported the medicinal properties of the different parts of the *C. nervosum* plant, this study provides the first pharmacological insight into the antidiabetic potential of the *C. nervosum* fruit. Ethanolic extract of *C. nervosum* fruit showed the highest antidiabetic capacity. This may, therefore, contain pharmacologically active and relatively non-toxic hypoglycemic and anti-adipogenic chemicals, which may be effective substitutes in the treatment of diabetes mellitus. The present findings would be useful for future research directions on the application of traditional medicinal plants in the development of nutraceuticals and pharmaceuticals.

## Figures and Tables

**Table 1:** Percentage yield of dried *Cleistocalyx nervosum* var. *paniala* fruit extracts.

Extract Used	Yield (%)
Distilled water	20.27 ± 2.11 ^c^
Ethanol	13.39 ± 1.29 ^b^
Hexane	8.01 ± 1.02 ^a^

Values are mean of three replicate determinations (*n* = 3) ± standard deviation. Mean values followed by different superscripts in a column are significantly different.

**Table 2:** In vitro α-amylase inhibitory activity of *C. nervosum* extracts.

Extract Used	α-Amylase Inhibitory Activity (IC_50_ mg/mL)
Distilled water	0.61 ± 0.09 ^c^
Ethanol	0.42 ± 0.07 ^b^
Hexane	no inhibitory activity
Acarbose (+ control)	0.09 ± 0.01 ^a^

Values are mean of three independent experiments (*n* = 3) ± standard deviation. Mean values followed by different superscripts in a column are significantly different.

**Table 3:** In vitro α-glucosidase inhibitory activity of *C. nervosum* extracts.

Extract Used	α-Glucosidase Inhibitory Activity (IC_50_ mg/mL)
Distilled water	0.44 ± 0.05 ^c^
Ethanol	0.23 ± 0.04 ^b^
Hexane	no inhibitory activity
Acarbose (+ control)	0.12 ± 0.02 ^a^

Values are mean of three independent experiments (*n* = 3) ± standard deviation. Mean values followed by different superscripts in a column are significantly different.

## Data Availability

Data is unavailable due to privacy restrictions.

## Supplementary Materials

The following supporting information can be downloaded at: https://www.mdpi.com/article/10.3390/plants12010112/s1, Figure S1: The cytotoxicity effect of water, ethanol and hexane extracts of C. nervosum at concentration of 0.5 mg/mL, 1.0 mg/mL, 1.5 mg/mL and 2.0 mg/ml on HepG2 after 72-h exposure assessed by MTT assay. Data are mean ± SD of three independent experiments; Figure S2: The cytotoxicity effect of water, ethanol and hexane extracts of C. nervosum at concentration of 0.5 mg/mL, 1.0 mg/mL, 1.5 mg/mL and 2.0 mg/ml on L6 myoblasts after 72-h exposure assessed by MTT assay. Data are mean ± SD of three independent experiments.
