# Supplementary material for: In Vitro Evaluation of Antidiabetic Potential of Cleistocalyx nervosum var. paniala Fruit Extract
Source: Plants (Basel). 2022 Dec 26;12(1):112. doi: 10.3390/plants12010112 (PMC9824712; doi:10.3390/plants12010112)
Supplement: Supplementary file 1 [file plants-12-00112-s001.zip › plants-2117002-supplementary.pdf]

Supplementary Materials

In vitro evaluation of antidiabetic potential of *Cleistocalyx nervosum var. paniala* fruit extract

Suttida Chukiatsiri[1]*, Nattakarn Wongsrangsap[1], Siriluk Ratanabunyong[1], Kiattawee Choowongkomon [1]

[1]Department of Biochemistry, Faculty of Science, Kasetsart University, Bangkok, Thailand

*Correspondence to: Suttida Chukiatsiri, Department of Biochemistry, Faculty of Science, Kasetsart University, 50 Ngam Wong Wan Road, Ladyao, Chatuchak, 10900, Bangkok, Thailand. E-mail: suttida.e@ku.th

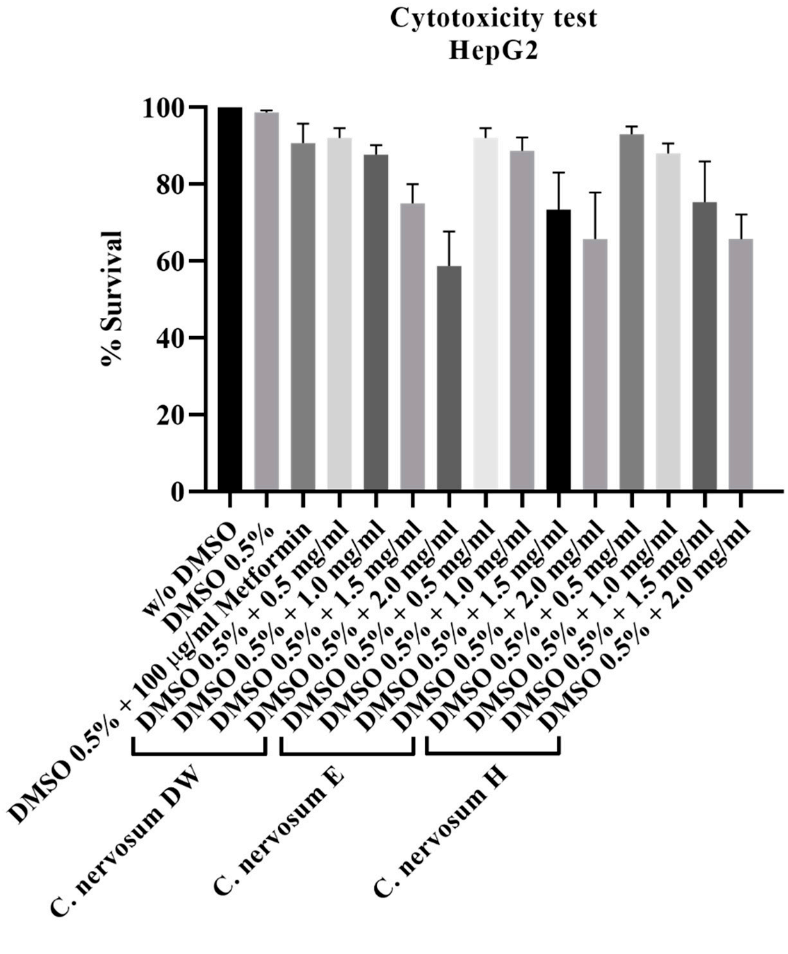

Figure S1. The cytotoxicity effect of water, ethanol and hexane extracts of *C. nervosum* at concentration of 0.5 mg/mL, 1.0 mg/mL, 1.5 mg/mL and 2.0 mg/ml on HepG2 after 72-h exposure assessed by MTT assay. Data are mean ± SD of three independent experiments.

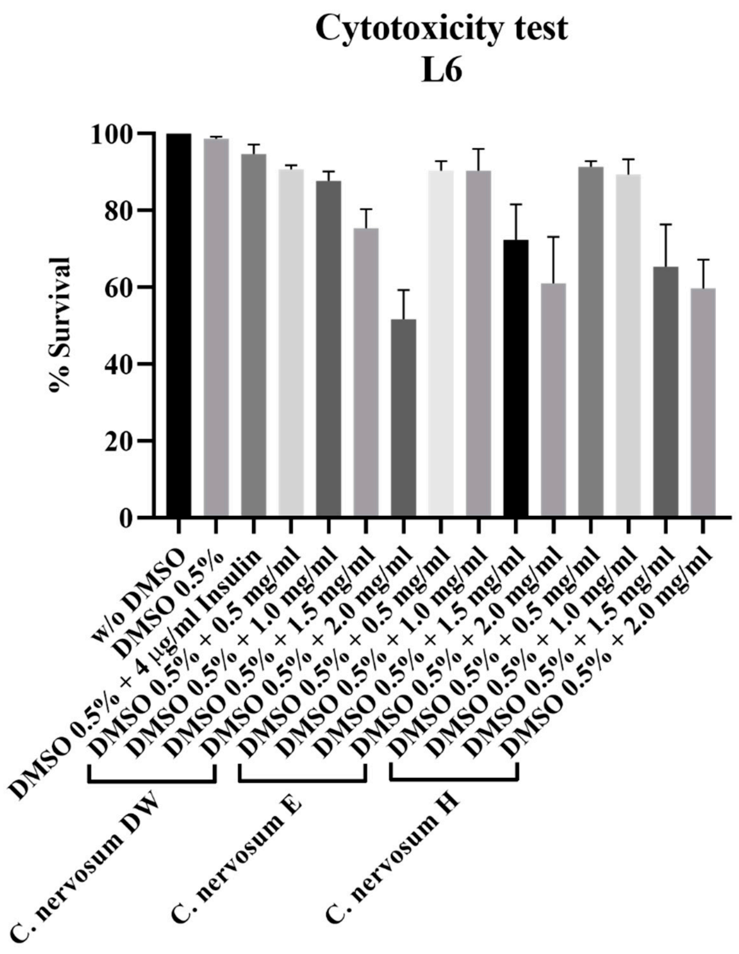

Figure S2. The cytotoxicity effect of water, ethanol and hexane extracts of *C. nervosum* at concentration of 0.5 mg/mL, 1.0 mg/mL, 1.5 mg/mL and 2.0 mg/ml on L6 myoblasts after 72-h exposure assessed by MTT assay. Data are mean ± SD of three independent experiments.
